# Translation and cultural adaptation of the Integrated Palliative care Outcome Scale including cognitive interviewing with patients and staff

**DOI:** 10.1186/s12904-017-0232-x

**Published:** 2017-09-11

**Authors:** Ingela Beck, Ulrika Olsson Möller, Marlene Malmström, Anna Klarare, Henrik Samuelsson, Carina Lundh Hagelin, Birgit Rasmussen, Carl Johan Fürst

**Affiliations:** 1 Institute for Palliative Care, Lund University and Region Skåne, Lund, Sweden; 2 Department of Health and Society, The Research Platform for Collaboration for Health, Kristianstad University, Kristianstad, Sweden; 3 Faculty of Medicine, Department of Clinical Sciences Lund, Lund University, Oncology, Lund, Sweden; 4 Department of Clinical Sciences Lund, Surgery, Lund University, Skåne University Hospital, Lund, Sweden; 5 Department of Health Sciences, Ersta Sköndal Bräcke University College, Stockholm, Sweden; 6 Palliative Care Unit, Ystad, Sweden; 7 Department of Care Science, Sophiahemmet University, Stockholm, Sweden; 8 Department of Neurobiology, Care Sciences and Society, Karolinska Institutet, Stockholm, Sweden; 9 Faculty of Medicine, Department for Health Sciences, Lund University, Lund, Sweden

**Keywords:** Patient-reported outcome measures, Outcome measurement, IPOS, Validity, Cognitive interviewing, Palliative care

## Abstract

**Background:**

To expand our clinical and scientific knowledge about holistic outcomes within palliative care, there is a need for agreed-upon patient-reported outcome measures. These patient-reported outcome measures then require translation and cultural adaptation, either from country-specific languages to English, or the other way around. The aim of this study was to translate and cross-culturally adapt the Integrated Palliative care Outcome Scale (IPOS) to the Swedish care context.

**Methods:**

Swedish versions of IPOS Patient and IPOS Staff were developed and culturally adapted using recommended guidelines including cognitive interviews with patients (*n* = 13) and staff (*n* = 15) from different care contexts including general and specialised palliative care.

**Results:**

The comprehension and judgement difficulties identified in the pre-final patient and staff versions were successfully solved during the cognitive interviewing process. IPOS was well accepted by both patients and staff, none of the questions were experienced as inappropriate, and all questions were judged important.

**Conclusions:**

In this study, we translated and culturally adapted the patient and staff versions of IPOS, and demonstrated face and content validity and acceptability of the scale through cognitive interviewing with patients and staff within residential care facility, surgical and specialised palliative home care units. Cognitive interviewing in parallel with patients and staff in rounds, with tentative analysis in between, was a suitable method for identifying and solving challenges with comprehension and evaluation in the pre-final version of IPOS. The Swedish IPOS is now available for use in a variety of clinical care settings.

**Electronic supplementary material:**

The online version of this article (10.1186/s12904-017-0232-x) contains supplementary material, which is available to authorized users.

## Background

To expand our clinical and scientific knowledge about holistic outcomes within palliative care (PC), there is a need for agreed-upon patient-reported outcome measures (PROMs). International agreement on these PROMs in PC would enable a systematic approach to assessing and collecting clinically relevant aspects of quality of life, as well as follow-up of treatment, care, and quality of care. PROMs require translation into specific languages as well as cultural adaptation in order to gain acceptable relevance for patients, family, and staff. The recommendations on outcome measures in PC are to use PROMs that capture the holistic nature of PC while remaining brief and straightforward and allowing for proxy reports [1]. The Integrated Palliative care Outcome Scale (IPOS; also referred to as the Integrated Patient care Outcome Scale) is intended to provide multidimensional perspectives on a patient’s situation, including physical, psychological, social, emotional, and spiritual concerns and needs. The health of PC patients deteriorates over time, and the cognitive impairment which frequently occurs results in problems with reporting outcomes. Assessment of PC needs is thus frequently dependent on proxy ratings, rating of patient’s symptoms/problems by another person, which have been shown to be useful [2], when patients are unable to self-report. Furthermore, an assessment tool in PC should be applicable both for research and for clinical practice [3]; that is, it should not burden the patient with assessments that are not relevant to their individual care.

IPOS is available, in both a patient (self-report) and a staff (proxy rating) version (IPOS Patient and IPOS Staff, respectively) for reporting outcome measures. IPOS Patient version should be used when patients are able to answer the questions, while the staff version allow proxy report when the patient is unable to self-report (1). IPOS was developed by integrating the most relevant items from the Palliative care Outcome Scale (POS) and POS-Symptom. POS has been used widely in clinical practice and research, is validated, and has shown good responsiveness to change [4]. IPOS has been developed and tested in both England and Germany [5].

The IPOS is comprised of 10 questions addressing patients’ concerns: symptoms, anxiety or low mood, family anxieties, overall feeling of being at peace, information needs, and practical concerns. The first question is an open question concerning patients’ main challenges. The second question is in the form of a list of 10 common symptoms, and includes space for three free options of individual symptoms to be added if needed. The questions are scored using a 0–4 Likert scale, with numerical and descriptive labels. IPOS Staff has one additional answer option, “cannot assess”, and the 10th item, *How did you complete this questionnaire?*, is excluded. The questions in IPOS Patient and IPOS Staff are given in Tables 2 and 3, respectively.

However, neither IPOS nor any other equivalent scale (i.e. with a holistic perspective including family aspects, a brief patient version, and a staff version for proxy use) is available for assessing patients’ needs within PC in Sweden. To ensure that an assessment measure is appropriate and to reduce the risk of inappropriate evaluations, both in clinical practice and research, it is important that the measure is validated [6, 7]. This study describes the cross-cultural adaptation process, for the Swedish versions of IPOS Patient and IPOS Staff. To assure face and content validity and enhance the quality of the final questionnaire, cognitive interviewing in the pre-testing phase is acknowledged as a prominent method [5, 8]. Cognitive interviewing involves administrating drafts of the questionnaire while collecting additional verbal information how subject comprehend, recall, and respond to the questions [9]. This to determine whether the question is generating the intended information and to reduce survey measurement errors [8]. This study describes the cross-cultural adaptation process for the Swedish versions of IPOS Patient and IPOS Staff equivalent to the source.

## Aim

The aim was to translate and cross-culturally adapt the Integrated Palliative care Outcome Scale to the Swedish care context.

## Methods

**Fig. 1:**
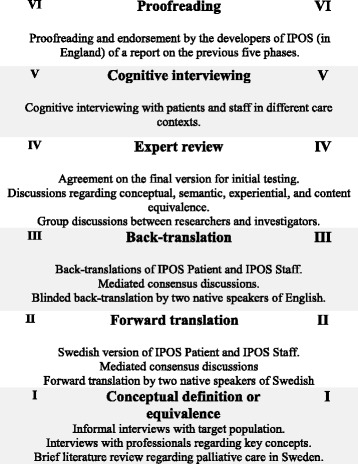
Overview of the phases in the translation and cultural adaptation process

Guidelines for the POS family of measures [10] were used for the translation and cross-cultural adaptation. These guidelines are based on commonly accepted translation and validation standard ideals (e.g. [11, 12]), and comprise six phases including pre-testing the pre-final version with cognitive interviewing and proofreading (Fig. 1). In brief, in phase I, conceptual definitions and equivalence of key concepts were identified by a literature review, interviews with staff, and informal interviews with the target population; here, three staff working in PC, three patients, and three relatives. The forward translation (phase II) was performed by two persons, one with clinical knowledge and one naïve in PC. A third person, independent and naïve in health care, acted as a mediator in a consensus discussion. This group generated a preliminary Swedish version of IPOS. The backward translation (phase III) was carried out by two persons working independently, one working in PC and one in curative health care. A third person, with knowledge of PC, was involved as mediator in consensus discussions. This group generated a back-translated version of the preliminary Swedish version of IPOS. The expert review (phase IV) was performed by researchers with knowledge in PC, two physicians and two registered nurses, and a journalist naïve in PC. This expert group met several times with the aim of evaluating, revising, and consolidating the instructions, items, and response format of the translated IPOS Patient and IPOS Staff, and developed the initial versions to be pre-tested with cognitive debriefing (interviewing; phase V, described in detail below). In the last phase (phase VI), the POS team in England proofread a written description of the previous phases. The present report will briefly describe phases I-IV and focus in more depth on the procedure and results from phase V.

### Cognitive interviews

Cognitive interviews were performed with patients and staff. The interviews comprised a combination of probing questions and an instruction to “think out loud” during completion of the questionnaire to generate verbal information [8]. An interview guide [cf.5] for probe questions was used, as well as probes surfacing during the interviews. As recommended in the literature [11] the interviews were carried out in rounds. In this study, there were three rounds with 8, 11 and 9 interviews, respectively. After each round, a tentative analysis of the interviews was carried out and a tentative revision of the IPOS patient and staff versions was made (by IB, UOM, MM, BR, and CJF) to be tested in the next round. Each interview was conducted in a place chosen by the participant, and was audio recorded. Field notes were made after each interview.

### Setting and participants for pre-testing IPOS by cognitive interviewing (phase V)

In order to include patients with PC needs as well as staff working with both general and specialised PC, participants were purposively sampled from three different care contexts, a residential care facility, a surgical inpatient unit and a specialised palliative home care unit, with the aim of achieving variation in relation to gender, age, diagnosis (malignant or non-malignant), registered nurses and assistant nurses. Inclusion criteria for patients were: 18+ years of age with clinically estimated PC or supportive care needs, and ability to give informed consent, answer the IPOS, and participate in an interview in Swedish. Inclusion criteria for staff were: working as assistant nurses or a registered nurse. One registered nurse at each unit selected 3–5 patients and staff based on the inclusion criteria, asked these individuals whether they were interested in participating, and provided a leaflet with information about the study. The contact details of those interested were passed on to the researchers, and each was approached by one of the researchers (IB, UOM, or MM).

### Analysis

The data were analysed using a thematic analysis [8] with the help of the NVivo 10 data software package. Audio files were imported and coded as patient or staff interviews, and parts that were important for the aim of the study were transcribed. Each interview was coded in sections: questions 1–9, any other symptoms, time frame, layout, and the overall impression of the IPOS. The results of patient and staff participants were compared and gathered for each question separately. One of the researchers (IB) had the main responsibility for the analysis, but the analysis took place in close collaboration with the other researchers (UOM, MM, BR, and CJF).

## Results

### Demographics

A total of 28 cognitive interviews were carried out between June and October 2015. Of the 29 potential participants approached, 28 (13 patients and 15 staff) gave their informed consent to participate, while one patient in the residential care facility declined participation. The patient interviews were 9–57 min long (median 24) and the staff interviews 18–70 min (median 33). The patients’ formal education level varied: elementary school (*n* = 5), upper secondary school (*n* = 7), and university/college (*n* = 1) (data not shown). Their median total sum score for IPOS was 21. Further demographic descriptions of the participants are given in Table 1.

**Table 1:** Demographics of the participants in the cognitive interviews (*n* = 28): patients (*n* = 13) and staff (*n* = 15) from different care contexts

	Residential care facility	Surgical unit	Specialised palliative care	Total
Patients	*n* = 4	*n* = 5	*n* = 4	*n* = 13
Age, range (median)	87–94 (90.5)	50–71 (55)	59–78 (72.5)	50–94 (70)
Gender, male (female)	1 (3)	2 (3)	2 (2)	5 (8)
Swedish as native language	4	4	3	11
Malignant (non- malignant) main diagnosis	0 (4)	3 (2)	4 (0)	7 (6)
^a^IPOS sum score, range (median)^b^	13–31 (13.5)	17–32 (25.5)	7–30 (28)	7–32 (21)
Staff	n = 5	n = 5	n = 5	n = 15
Age, range (median)	34–57 (38.5)	25–60 (40)	33–60 (38)	25–60 (39)
Gender, male (female)	0 (5)	1 (4)	2 (3)	3 (12)
Swedish as native language	5	5	5	15
RN (NA)	3 (2)	3 (2)	5 (0)	11 (4)
^a^IPOS sum score, range (median)^b^	13–33 (22)	22–44 (31)	9–31 (31)	9–44 (31)

^a^IPOS sum score range 0–68 (without scores for other symptoms)

^b^The patient IPOS sum scores and the staff IPOS sum scores do not apply to the same patients

### Phases I - IV

#### Phase I

The brief literature review and the initial interviews with the target population showed a common language with regard to definitions and terminology in PC. The IPOS questions were regarded as important, relevant, and generally easy to understand and answer.

#### Phase II

There were few obvious discrepancies in the forward translations. When the translators had chosen different terms, the options were discussed and negotiated. The main situations where this occurred were for the terms *at peace* and *addressed* (Q6 and Q9); these have no equivalent terms in Swedish, and so the Swedish terms for *satisfied* and *met* were chosen (see Additional file 1).

#### Phases III-IV

The back-translations were compared, and no faulty or incorrect translations were discovered; only minor grammar discrepancies, which were adjusted. However, the expert review did discuss whether the translation of *depressed* should use the Swedish term for *depressed* (i.e. the medical diagnosis) or the Swedish term for *gloomy* (i.e. how people talk in everyday interactions); the term for *depressed* was chosen. The group agreed on proposed Swedish versions of IPOS Patient and IPOS Staff to be pre-tested.

#### Phases V and VI

Findings from the analyses of the cognitive interviews are presented under three headings: *comprehension*, *judgement*, and *acceptance*. Adjustments were based on difficulties identified due to comprehension and judgement (Tables 2 and 3). The final IPOS patient and staff versions were endorsed by the original developers after proofreading the report of the entire process.

**Table 2:** Issues regarding IPOS questions identified in cognitive interviews with patients (*n* = 13), and items revised

Items in the English version	Patients’ comprehension of the pre-final Swedish IPOS Patient	Question revised
Q1. What have been your main problems or concerns over the past 3 days?	Good comprehension overall. All patients except one specified 1–3 main problems or concerns.	No
Q2. Below is a list of symptoms, which you may or may not have experienced. For each symptom, please tick one box that best describes how it has affected you over the past 3 days.	One patient had to read the question several times (1/6). A revision with the first sentence removed was tested (*n* = 9), and comprehension was then good (9/9). Some patients (3/13) did not consider three days to be long enough; one stated that the past week would have been a better time window.	Yes
Pain	Some patients (2/13) found it hard to judge the severity of pain and how it affected them, as this symptom fluctuated over the three days. They solved this problem by estimating a mean value over the three days.	No
Shortness of breath	Good comprehension overall (12/13). One thought the Swedish term^a^ for breathlessness was a difficult word.	Yes
Weakness or lack of energy	Overall good comprehension except by one patient who did not understand the difference between these two terms.	No
Nausea (feeling like you are going to be sick)	Good comprehension by all patients.	No
Vomiting (being sick)	Good comprehension by all patients.	No
Poor appetite	Good comprehension by all patients. Judging the severity was difficult for one patient, as she was tube fed.	No
Constipation	Good comprehension by all patients.	No
Sore or dry mouth	Good comprehension overall (12/13). One patient considered that it was not possible to have pain in the mouth.	No
Drowsiness	Good comprehension overall (11/13). One patient did not understand what drowsiness was, and some thought it also could be something positive.	No
Poor mobility	Good comprehension by all patients.	No
Please list any other symptoms not mentioned above, and tick one box to show how they have affected you over the past 3 days.	One patient got stuck on this instruction, and had to read it several times in order to understand it. A revision was tested (n = 9), and showed good comprehension (9/9).	Yes
Over the past 3 days:		
Q3. Have you been feeling anxious or worried about your illness or treatment?	Good comprehension by all patients.	No
Q4. Have any of your family or friends been anxious or worried about you?	Good comprehension (6/6). However, the term for friends is not commonly used in the Swedish care context. The Swedish term for next-of-kin was tested as a replacement (n = 7), and showed good comprehension. This term includes not only relatives (e.g. sisters, first cousins) but also close friends (e.g. neighbours).	Yes
Q5. Have you been feeling depressed?	Some patients (4/13) interpreted the Swedish term for depressed as a diagnosis, and so the Swedish term for gloomy was tested as a replacement (*n* = 11). All patients either preferred the Swedish term for gloomy or had no preference between the two terms.	Yes
Q6. Have you felt at peace?	Some patients (2/13) understood the question with the Swedish term for at peace (i.e. satisfied) as asking whether they were satisfied with their care or their achievements, and not the intended meaning of spiritual wellbeing. Replacement terms were tested. Some patients (4/13) connected the Swedish term for inner peace with being religious. The Swedish term for calmness and stillness within themselves was the one most patients (7/13) used when they talked about inner peace or matters related to spiritual wellbeing, such as accepting their situation.	Yes
Q7. Have you been able to share how you are feeling with your family or friends as much as you wanted?	Some patients (3/13) misunderstood the question as asking whether they wanted to share how they were feeling with family or friends, and one had difficulty understanding the Swedish term for being able to share. One patient said that she did not share how she felt with her friends. The term for next-of-kin was tested as a replacement (see question 4).	Yes
Q8. Have you had as much information as you wanted?	Some patients (5/13) wondered what information this question referred to, and from whom. Others understood that the question referred to information about their disease, their situation, or the care. One patient did not answer the question.	Yes
Q9. Have any practical problems resulting from your illness been addressed? (such as financial or personal)	Most of the patients (12/13) spent time reading and rereading this question, and had some trouble understanding what practical problems were referred to. The example in parentheses was helpful for some, but seemed to be more confusing for others.	Yes
Q10. How did you complete this questionnaire?	Good comprehension by all patients.	No

^a^The Swedish terms are shown in Additional file 1

**Table 3:** Issues regarding IPOS questions identified in cognitive interviews with staff (*n* = 15), and items revised

Items in the English version	Staff comprehension of the pre-final Swedish IPOS Staff	Question revised
Q1. What have been the patient’s main problems or concerns over the past 3 days?	Good comprehension by all staff.	No
Q2. Please tick one box that best describes how the patient has been affected by each of the following symptoms over the past 3 days?	Some staff (3/15) considered 3 days too short, and one (1/15) considered it too long. Some (2/15) thought this timescale was best for first-time visits and in end-of-life care.	No
Pain	Good comprehension by all staff. Judging the severity of pain and how this affected the patient was difficult for some staff (2/15), as it varied over time.	No
Shortness of breath	Good comprehension overall (14/15). One staff member suggested using the Swedish term^a^for shortness of breath instead of the Swedish term for breathlessness.	No
Weakness or lack of energy	One staff member interpreted the Swedish term for weakness or lack of energy as meaning lack of nutrition. The Swedish term for feebleness was tested as a replacement (9/15), and found to be associated with low general condition.	No
Nausea (feeling like you are going to be sick)	Good comprehension by all staff.	No
Vomiting (being sick)	Good comprehension by all staff.	No
Poor appetite	Good comprehension by all staff.	No
Constipation	Good comprehension by all staff.	No
Sore or dry mouth	Good comprehension by all staff. One staff member thought that mucus in the oral cavity was not included in this symptom, but had no suggestions for amendments.	No
Drowsiness	Some staff interpreted this as referring to the patient being asleep (3/15), or the patient falling asleep due to being affected by drugs (3/15). One felt that it was difficult to distinguish between drowsiness and weakness or lack of energy.	No
Poor mobility	Good comprehension by all staff.	No
Please list any other symptoms and tick one box to show how you feel each of these symptoms has affected the patient over the past 3 days.	One staff member found the layout confusing in that the instruction text disrupted the row of text with the answer options. A revision was tested (n = 11) with good comprehension (11/11).	Yes
Over the past 3 days:		
Q3. Has s/he been feeling worried about his/her illness or treatment?	Good comprehension overall (14/15). One staff member was unsure what the question referred to. A gender-neutral Swedish pronoun (equivalent to singular “they”) was tested as a replacement for he/she, but none of the staff found this preferable.	No
Q4. Have any of his/her family or friends been anxious or worried about the patient?	Good comprehension by all staff. The Swedish term for next-of-kin was tested as a replacement for the Swedish for family or friends (n = 2). The term for next-of-kin was interpreted as meaning a family member, a close friend, a neighbour, or even, a pet.	Yes
Q5. Do you think s/he felt depressed?	The Swedish term for gloomy was tested as a replacement for the Swedish term for depressed (n = 15), and almost all staff (12/15) preferred the term for gloomy, as it was considered to be more inclusive and not associated with a diagnosis. Some staff (2/15) thought it was not clear who the question was referring to.	Yes
Q6. Do you think s/he has felt at peace?	Some staff (n = 2/15) interpreted the question with the Swedish term for at peace (i.e. satisfied) as mainly asking if the patient was satisfied with the care and their encounter with the staff. Replacement terms were tested. The Swedish term for inner peace was interpreted by some staff (3/15) as being associated with death and end of life, and by others (2/15) as having a religious connection.	Yes
Q7. Has the patient been able to share how s/he is feeling with his/her family or friends as much as s/he wanted?	Some staff (5/15) thought the question was unclear. Three interpreted the question as asking if the patient had the ability to talk with someone (e.g. the ability to make a phone call to someone). The Swedish term for being able was problematic. The term for next-of-kin was tested (n = 2) as a replacement for family or friends, and (2/2) was interpreted as meaning a family member or a close friend.	Yes
Q8. Has the patient had as much information as s/he wanted?	Good comprehension overall (14/15). One staff member initially had difficulty understanding what kind of information the question was about, but after a short while interpreted it as a wide question.	No
Q9. Have any practical problems resulting from his/her illness been addressed? (such as financial or personal)	Several staff (6/15) perceived the Swedish term for addressed (i.e. met) as being difficult in a question about practical problems, as it does not imply any practical help. Some (3/15) thought that the text in the parentheses was confusing and restrictive.	Yes

^a^The Swedish terms are shown in Additional file 1

### Comprehension

Some comprehension difficulties were identified in both the patient and staff interviews (Q1, Q2, and Q6) regarding the terms for *shortness of breath*, *drowsiness*, *at peace*, and *depressed* (see Tables 2 and 3). Various replacements for these terms were tested, and the questions were adjusted to use the terms that were considered most appropriate. In addition, comprehension difficulties due to lengthy sentences were identified (Q7 and Q9), and were solved by simplifying the sentences and putting subordinate paragraphs in brackets. For detailed information on how the participants comprehended each question, see Tables 2 and 3.

### Judgement

The time window for the questions (*over the past three days*) was considered too short for some patients at the residential care facility and too long for some at the surgical unit. Some staff in specialised PC experienced the time window as too short for stable patients with weekly visits. The answer option *overwhelmingly* was identified as unnatural to use for assessing symptoms (Q2). The Swedish term for *worst possible* was tested as a replacement; this worked well and was used in the adjusted version. In some cases, it was difficult for both patients and staff to assess how symptoms such as *pain* had affected the patient over time, as the symptom fluctuated. However, no adjustment was made. Another concern was related to the response options for Q9; as each response option was long and included the Swedish term for *meet* (as in *meeting needs*), the options were simplified and this term was replaced with the Swedish term for *help*. For detailed information on these concerns of judgement, see Tables 2 and 3.

### Acceptance and applicability

IPOS, as a whole, was well accepted by both patients and staff. No question was experienced as inappropriate, and all questions were judged important. Some staff stated that the questions were similar to everyday questions used in their clinical practice, but here used in a structural way. Patients spontaneously expressed that some of the questions were very good, such as the opportunity to list other symptoms (Q2), and the question about anxiety or worry over their illness or treatment (Q3); when discussing this latter question, some patients started to talk about the future with their relatives as well as about death and dying. The patients also spontaneously stated that the questions about sharing their feelings (Q7) and practical problems (Q9) were very important. The staff, too, spontaneously expressed the opinion that some of the items were very good; specifically, the questions about whether the patient had felt at peace (Q6), whether the patient had as much information as they wanted (Q8), and practical problems (Q9). After completing the IPOS versions, both patients and staff stated that the answers gave an understanding of the patient’s total situation. It is however worth noting that some of the staff had difficulty taking a patient perspective when completing the questionnaire, and had to be reminded during the interview.

The time to complete IPOS and perform the interview was regarded as acceptable by both patients and staff, and as a whole, the questionnaire was experienced as easy to complete. In fact, the patients stated that it was important to have the possibility to talk with someone about their situation during IPOS completion, and they felt that the time invested was worthwhile. Some patients expressed concerns regarding how the staff would use IPOS, for example asking if it would make any difference. Some staff were also concerned about how to use IPOS to make a difference for the patient, whereas others considered it a helpful tool to get a holistic view of the patient’s situation that could be used as a foundation for planning care.

## Discussion

In this study, we translated and culturally adapted IPOS Patient and IPOS Staff, and demonstrated face and content validity and acceptability of the scale through cognitive interviews with patients and staff within, residential care facility, surgical and specialised palliative care units. The Swedish translation of IPOS has thus been shown to be acceptable for both patients and staff. However, there were certain concepts where a direct translation from English to Swedish became misleading and in need of cultural adaptation; the cognitive interviews with patients and staff proved invaluable for this adaptation.

The cognitive interviews showed that the pre-final versions of IPOS Patient and IPOS Staff were generally well-comprehended. Nevertheless, there were some difficulties related to comprehension and validation in the pre-final versions, which were solved with refinements during phase V. We used the guidelines [10] recommended for cross-cultural adaptation to be able to create a version of the original instrument in a target language conceptually equivalent to the source instrument. As shown in this study, it is important to perform cognitive interviews (phase V) in order to reveal and further explore discrepancies in the pre-final version. Cognitive interviews with individuals in the intended target population offer one approach to investigate inconsistencies in the pre-final versions of instruments [7]. Similarly positive results from cognitive interviews (i.e. identifying and solving challenges with comprehension and evaluation regarding PC outcome measurements) have been described before [5]. According to Streiner and Norman [7], if the cognitive interviews do not uncover any inconsistencies in the pre-final version, this is most likely a sign of problems with the interview process.

The main problems with comprehension in the pre-final version of IPOS involved finding an appropriate Swedish term for *at peace*. The replacement terms tested were either understood as meaning satisfaction with care or were only appropriate for those that practiced religion, and hence not appropriate for some patients and staff. The intention of the question is to measure spiritual wellbeing [5]. One reason for the difficulty finding an appropriate term in Swedish could be that Sweden is described as one of the most secular and individualistic societies in the world [13]. We eventually found that both patients and staff considered the Swedish term for *calmness and stillness within themselves* to be a suitable expression for *feeling at peace* in terms of spiritual wellbeing, without excluding either those that practice religion or those who do not.

Another problem with comprehension was identified for *drowsiness*, since this was not seen as a purely negative feeling, either as experienced by the patients or as perceived by the staff. Patients with hormone-refractory prostate cancer have described drowsiness as a pleasant feeling [14]. In IPOS, drowsiness is rated on the basis of how it affects the patient in a negative way. If drowsiness is experienced as a pleasant feeling, it should be scored as *not at all*. However, it is important to be aware that experiences of drowsiness could change over time, from a negative feeling to a positive (17). There were also inconsistencies related to the Swedish term for *addressed* in the question about practical problems (Q9). Both patients and staff considered this question to be an important one, but it seemed to be important that the patients got actual help with a practical problem, rather than simply having an opportunity to discuss it. The final version included the Swedish term for *help* instead of *addressed*, which is more solution-focused.

The response options and references to time generally worked well, but some inconsistencies were identified. The time reference of the past three days was not suitable for all participants; for some it was too short and for some too long. The wish for a longer time frame was also found among some of the patients in a previous study [5], and there is another version of IPOS which uses a recall period of seven days. One of the response options for scoring symptoms, the Swedish term for *overwhelmingly*, is problematic to use in weighing symptoms, as it could mean something positive. Other scales have used the term *worst possible* as an alternative for the highest value [15, 16], and this was found to also work well here.

Both IPOS Patient and IPOS Staff were well accepted. All patients, regardless of level of PC needs, age, education level, or care context, accepted the time and energy invested in completing IPOS within a cognitive interview. It even seemed that the patients valued the opportunity to answer and talk about the aspects and issues in IPOS, provided that the staff listened and tried to make a difference for the patient. It is important that an outcome measure in palliative and end of life care research captures clinically relevant data and is easy to administer across care settings [17], and IPOS appears to have both of these properties. However, the patients highlighted the importance of being able to talk about their concerns and having their needs met. It is important not to overload patients, but it is also important not to underestimate their wishes, for example their desire to talk about their situation when completing a PROM.

Questions that the patients especially appreciated concerned individual symptoms as well as psychosocial and practical concerns. The patients highlighted the same questions as the staff; that is, the questions concerning the addressing of practical problems, spiritual wellbeing, and the need for information. Thus, IPOS seems to include appropriate questions for patients in need of holistic PC. One reason for highlighting these questions could be that non-physical aspects are easily overlooked and physical symptoms are prioritized [18], and so these areas emerge as important for both patients and staff. It is crucial for a holistic PC to use measurement tools that include dimensions other than physical symptoms, to ensure that other concerns that might not naturally be addressed are acknowledged [19]. PROMs such as IPOS are important tools for systematic assessment of distressing problems, symptoms and needs among patients. It is however necessary to understand that there may be issues beyond those contained in any one instrument, and so there is a need for expertise in introducing the instrument, in discussing areas in detail, and in being sensitive to issues outside the instrument. The Swedish IPOS is now available for use in a variety of clinical settings. The next step, to further contribute to increased knowledge about holistic outcomes within PC, is to psychometrically validate Swedish IPOS, patient and staff versions.

## Methodological discussion

To our knowledge, this is the first study showing that simultaneous interviews with patients and staff to pre-test patient and staff versions of a PROM can provide supplementary information for cultural adaptation. It was an advantage to conduct cognitive interviews in parallel with patients and staff, as this gave broader perspectives as a basis for improving and elucidating the Swedish-language IPOS. We consistently refrained from making changes in the IPOS patient and staff versions without support from the patient and staff interviews respectively.

Another advantage was conducting the cognitive interviews successively, with tentative analysis of the interviews in between. This process allowed us to replace problematic terms and sentences and test them before the final revision. Cognitive interviews in rounds of between 5 and 15 interviews each is described as a useful approach, but it is not clear how many rounds are needed [8]. We conducted three rounds with roughly 10 participants in each, and found these especially helpful in choosing an appropriate Swedish term for *at peace*.

The sample size could be considered a limitation of this study. We initially interviewed three patients, three relatives, and three staff in order to investigate the most crucial concepts, and in the second stage we interviewed a total of 13 patients and 15 staff. The recommended sample size in this type of interview method varies from 5 to 15 participants [7, 10] up to 30–40 [11]. Some researchers imply that the number should be based on information redundancy, which in most cases results in 8–15 participants [7]. In total, 37 persons of the target population were interviewed at one stage or another during the process of translation. Only two of the patients, and none of the staff, had a native language other than Swedish. The recommendation is to perform interviews with native speakers fluent in the target language [10], but also to include people who are similar to the intended respondents [7]. We recommend caution when transferring these results to groups other than native Swedish speakers.

## Conclusions

In this study, we translated and culturally adapted IPOS Patient and IPOS Staff for a population of Swedish patients, and demonstrated the face and content validity and acceptability of the scale through cognitive interviewing with patients with supportive and PC needs as well as staff working with supportive care, general PC, and specialised PC. Recommended validation processes were used to create a version conceptually equivalent to the source. IPOS Patient and IPOS Staff are now available to use clinically in various care settings in Sweden, early in the palliative care phase as a PROM as well as late in end-of-life care as a proxy. The scene is set for an expansion of our knowledge about holistic outcomes, both clinically and scientifically, within palliative care in Sweden.

## Additional file

Additional file 1: The Swedish terms corresponding to the English terms in the Results section and Tables 2 and 3, in alphabetic order. (DOCX 19 kb)

## Abbreviations

IPOS: Integrated Palliative care Outcome Scale/Integrated Patient care Outcome Scale
PC: Palliative care
PROM: Patient-reported outcome measure
Q: Question

## References

[1] Bausewein C, Daveson BA, Currow DC, Downing J, Deliens L, Radbruch L, Defilippi K, Lopes Ferreira P, Costantini M, Harding R, et al. EAPC white paper on outcome measurement in palliative care: improving practice, attaining outcomes and delivering quality services - recommendations from the European Association for Palliative Care (EAPC) task force on outcome measurement. Palliat Med. 2015; 10.1177/0269216315589898 26068193

[2] Kutner JS, Bryant LL, Beaty BL, Fairclough DL (2006). Symptom distress and quality-of-life assessment at the end of life: the role of proxy response. J Pain Symptom Manag.

[3] Higginson IJ, Evans CJ, Grande G, Preston N, Morgan M, McCrone P, Lewis P, Fayers P, Harding R, Hotopf M (2013). Evaluating complex interventions in end of life care: the MORECare statement on good practice generated by a synthesis of transparent expert consultations and systematic reviews. BMC Med.

[4] Collins ES, Witt J, Bausewein C, Daveson BA, Higginson IJ, Murtagh FE. A systematic review of the use of the palliative care outcome scale (POS) and the support team assessment schedule (STAS) in palliative care. J Pain Symptom Manag. 2015; 10.1016/j.jpainsymman.2015.07.015 26335764

[5] Schildmann EK, Groeneveld EI, Denzel J, Brown A, Bernhardt F, Bailey K, Guo P, Ramsenthaler C, Lovell N, Higginson IJ (2016). Discovering the hidden benefits of cognitive interviewing in two languages: the first phase of a validation study of the integrated palliative care outcome scale. Palliat Med.

[6] Polit DF, Beck CT (2010). Generalization in quantitative and qualitative research: myths and strategies. Int J Nurs Stud.

[7] Streiner DL, Norman GR (2008). Health measurement scales: a practical guide to their development and use.

[8] Beatty PC, Willis GB (2007). Research synthesis of cognitive interviewing. Public Opin Q.

[9] Willis GB (2005). Cognitive interviewing a tool for improving questionnaire design.

[10] The Palliative care Outcome Scale family of measures; Manual for cross-cultural adaptation and psychometric validation [http://pos-pal.org/Resources.php].

[11] Beaton DE, Bombardier C, Guillemin F, Ferraz MB (2000). Guidelines for the process of cross-cultural adaptation of self-report measures. Spine.

[12] Guillemin F, Bombardier C, Beaton D (1993). Cross-cultural adaptation of health-related quality of life measures: literature review and proposed guidelines. J Clin Epidemiol.

[13] Welzel- Ingleharts Cultural Map [http://www.worldvaluessurvey.org/WVSContents.jsp].

[14] Lindqvist O (2011). Living with bodily changes in hormone-refractory prostate cancer. Semin Oncol Nurs.

[15] Watanabe SM, Nekolaichuk CL, Beaumont C (2012). The Edmonton symptom assessment system, a proposed tool for distress screening in cancer patients: development and refinement. Psychooncology.

[16] Henoch I, Axelsson B, Bergman B (2010). The assessment of quality of life at the end of life (AQEL) questionnaire: a brief but comprehensive instrument for use in patients with cancer in palliative care. Qual Life Res.

[17] Evans CJ, Benalia H, Preston NJ, Grande G, Gysels M, Short V, Daveson BA, Bausewein C, Todd C, Higginson IJ (2013). The selection and use of outcome measures in palliative and end-of-life care research: the MORECare international consensus workshop. J Pain Symptom Manag.

[18] Bahrami M, Arbon P (2012). How do nurses assess quality of life of cancer patients in oncology wards and palliative settings?. Eur J Oncol Nurs.

[19] Hagelin CL, Wengstrom Y, Tishelman C, Furst CJ (2007). Nurses' Experiences of clinical use of a quality of life instrument in palliative care. Contemp Nurse.

