# Supplementary material for: Translation and cultural adaptation of the Integrated Palliative care Outcome Scale including cognitive interviewing with patients and staff
Source: BMC Palliat Care. 2017 Sep 11;16:49. doi: 10.1186/s12904-017-0232-x (PMC5594532; doi:10.1186/s12904-017-0232-x)
Supplement: Additional file 1: — The Swedish terms corresponding to the English terms in the Results section and Tables 2 and 3, in alphabetic order. (DOCX 19 kb) [file 12904_2017_232_MOESM1_ESM.docx]

| Additional file 1. The Swedish terms corresponding to the English terms in the Results section and Tables 2 and 3, in alphabetic order. | |
| --- | --- |
| **English term** | **Swedish term** |
| Breathless | Andfådd |
| Be able | Kunna |
| Calmness and stillness within themselves | Lugn och ro inombords |
| Depressed | Deprimerad |
| Drowsiness | Dåsighet |
| Feeble | Orkeslös |
| Gloomy | Nedstämd |
| Help | Hjälp |
| Inner peace | Inre frid |
| Met | Bemötts |
| Next-of-kin | Närstående |
| Overwhelmingly | Överväldigande |
| Satisfied | Tillfreds |
| Share | Dela |
| S/he | Hen |
| Shortness of breath | Andnöd |
| Weakness or lack of energy | Svaghet eller brist på energi |
| Worst possible | Värsta tänkbara |
